# Perspectives on Aging and Quality of Life

**DOI:** 10.3390/healthcare11152131

**Published:** 2023-07-26

**Authors:** Shinichi Noto

**Affiliations:** Department of Rehabilitation, Niigata University of Health and Welfare, Niigata 9503198, Japan; noto@nuhw.ac.jp

**Keywords:** aging, older adults, lifestyle-related diseases, quality of life

## Abstract

The aging of the world’s population and the health problems accompanying it are becoming increasingly severe. Healthcare policies in developed countries focus on how to prevent and treat diseases associated with aging and how to maintain quality of life. Typical age-related diseases include deafness, cataracts, osteoarthritis, chronic obstructive pulmonary disease, diabetes mellitus, and dementia. Although the mechanisms by which these diseases develop differ, they are all caused by the accumulation of molecular and cellular damage over time. In addition, age-related diseases can cause a decline in physical and mental functions and the ability to perform activities of daily living, as well as the loss of roles in society and a sense of fulfillment in life. Therefore, there is a need for treatment and measures to accurately grasp and maintain quality of life. This review aims to introduce areas and representative papers expected to be contributed to the special issue of “Aging and Quality of Life”.

## 1. Introduction

An aging population has various implications on health, which in turn has a significant impact on medical care and health policy. According to the World Health Organization (WHO) [1], by 2030, one in six people worldwide will be aged 60 years or older. Furthermore, the population aged 60 years and older is expected to increase from 1 billion in 2020 to 1.4 billion in 2030. In addition, by 2050, this population is expected to double (2.1 billion). Similarly, the number of people aged 80 years and older is expected to triple between 2020 and 2050 and reach 426 million. This shift in population distribution toward older adults, known as population aging, began in high-income countries. However, it is most advanced in Japan, where 30% of the population is already aged 60 years or older. Moreover, this aging has further extended to low- and middle-income countries. It is estimated that by 2050, two-thirds of the world’s population aged 60 and older will live in low- and middle-income countries. The effects of this aging population are causing great concern for human health, and society requires major changes. Of particular importance is the question of how to stay healthy during an extended life expectancy. This is because we desire to live in good health for as long as possible.

According to the Organization for Economic Co-operation and Development (OECD) [2], the average life expectancy after 65 years in member countries was approximately 20 years. Furthermore, it was the longest for Japanese women (25 years). The average life expectancy is increasing every year and has the advantage of providing opportunities to pursue new activities, such as further education, new careers, and long-neglected passions. However, it also leads to the emergence of several complex health conditions, commonly known as geriatric syndromes. The most common of these health conditions is frailty, a syndrome characterized by reduced functional capacity and increased vulnerability to disease. It is associated with various adverse health outcomes, including mortality, falls, fractures, and institutionalization [3]. Another typical health condition of geriatric syndromes is sarcopenia, estimated to affect 10% to 16% of the world’s older adults. It is an old condition characterized by progressive muscle mass and function loss and is associated with various adverse health outcomes [4].

However, how meaningful the remainder of one’s life is after aging is based on the maintenance of good health. Hence, it is important to avoid falling sick and to maintain or improve one’s quality of life even when one falls sick. The challenge for those involved in geriatric and community health care is to prevent the development of geriatric syndromes in the aging population and to take approaches to improve health-related quality of life (HRQOL), even when such conditions do occur.

In this paper, I first review the biological effects of aging. Then, diseases that are more likely to occur with aging are summarized, and approaches to these diseases are introduced. Then, the impact of these medical approaches and community services on HRQOL of the older adults is reviewed. Through this review, I intend to provide a comprehensive overview of the health issues associated with aging and how important it is to maintain or improve health-related quality of life even when health conditions deteriorate. I hope this review will raise more professional questions about health care and community services for older adults and, above all, how they can improve their HRQOL.

## 2. Negative Effects of Aging on Health

At the biological level, aging results from the accumulation of molecular and cellular damage over time. This results in a gradual decline in physical and mental capacities and an increase in the risk of disease. In addition to biological changes, retirement, loss of purpose in life, relocation to more suitable housing, and the death of a friend or partner also often result in psychological damage. In addition to physical and mental decline, social restrictions also result in a diminished quality of life.

### 2.1. Cellular Changes

First, with aging, the oxygen supply to all organs and tissues and the partial pressure of oxygen in tissues decrease, resulting in hypoxia. It is then thought to be involved in the development of inflammatory diseases, tumors, and cardiac disease [5]. Excessive production of reactive oxygen species also leads to the destruction of nucleic acids and proteins, thereby altering cellular structures and functional outcomes. The detrimental effect on the organism caused by such oxidative reactions is called oxidative stress. It causes aging and chronic degenerative diseases such as cardiovascular disease, diabetes mellitus, and chronic kidney disease, Alzheimer’s disease (AD), Parkinson’s disease, and other neurodegenerative diseases [6].

Advanced glycation end-products (AGEs) is a general term for heterogeneous derivatives produced by non-enzymatic reactions of reducing sugars with proteins. It has recently been suggested that AGEs are involved in the pathogenesis of chronic hyperglycemia and age-related diseases. The accumulation of AGEs and their derivatives can modify proteins and could promote aging by activating several inflammatory signaling pathways via AGEs-specific receptors [7].

### 2.2. Chromosome Changes

Telomeres are structures at the ends of chromosomes that shorten with each cell division. Shortening of telomeres, which are specialized nucleoprotein structures at the ends of linear chromosomes, has been linked to aging [8]. When telomeres do not function, chromosomes lose their protective structure, and fusion and breakage phenomena occur, leading to further genomic instability such as cell arrest and death [9]. Additionally, impairment of the telomere function, coupled with impairment of the senescence/apoptosis response, causes chromosomal instability [10].

### 2.3. Psychological Changes

In addition to the above-mentioned physiological changes, various age-related events negatively impact mental health. Loss of a role or purpose in life after retirement, the independence of children, or the death of a partner or good friend can cause psychological harm [11,12]. Except for those who can retire with sufficient savings, many older adults could experience financial hardship due to a decrease in income [13]. It is easily perceived that these factors can negatively impact health.

As described above, as we age, cells and chromosomes degenerate, and mental factors also contribute to the development of various diseases. In the next section, these factors are examined in detail.

## 3. Diseases Likely to Occur with Age

This section describes various diseases that are more likely to develop with aging and their impact on HRQOL of older adults. Hearing loss, cataracts, back and neck pain, osteoarthritis, osteoporosis, chronic obstructive pulmonary disease, diabetes, depression, and dementia are common problems in older adults. Older adults are also more susceptible to lifestyle-related diseases, such as stroke, coronary artery disease, and cancer. Furthermore, as people age, they are more likely to experience multiple diseases simultaneously.

Hearing loss is the third most common chronic health condition that affects older adults. Age-related hearing loss affects one in three adults over the age of 65 years. Sensorineural hearing loss related to loss of outer hair cells was reported to be present in most adults patients aged 85 years and older [14]. It is caused by exogenous and endogenous factors, such as heredity, aging, and exposure to noise and toxins [15]. Abraham et al. [16] also reported the danger of hiding hearing-impaired in older adults among patients with dementia.

Cataract is the most common cause of blindness worldwide. A report stated that more than 70% of people over the age of 80 years had clinically significant age-related cataracts [17]. Another study reported that cataract patients had an increased risk of dementia if they did not undergo surgical treatment [18]. There is no doubt that cataract is a serious disease, as it has been reported that older adults are more susceptible to the impact of cataracts on their HRQOL [19].

Osteoarthritis is another common cause of disease in the aging population. Joints of the lower extremities, which generally bear most of the body weight, are most often affected by osteoarthritis. In particular, knee osteoarthritis is a presumed risk factor for several medical conditions, including cardiovascular disease and falls [20]. In contrast, degenerative changes in the joints of the upper extremities are equally common, predisposing to osteoarthritis in shoulder, elbow, and hand joints [21].

Osteoporosis is a bone metabolic disease that women are typically at higher risk of developing than men. A reason for the higher incidence of osteoporosis in women is that production of estrogen by their ovaries becomes variable during perimenopause and ceases post-menopause. Notably, estrogen promotes the activity of osteoblast cells that produce bone [22]. Osteoporosis is also a predisposing factor for increased risk of hip, spine, and other skeletal site fractures; the clinical impact and economic burden of such injuries has contributed towards advocacy for periodic physical examinations for older women that prompt appropriate interventions [23].

Chronic obstructive pulmonary disease (COPD) is the third leading cause of death worldwide. The greatest risk factors include smoking and aging. COPD develops after the age of 60 years and its pathogenesis is characterized by fibrosis and remodeling of the small airways, accompanied by destruction of the lung parenchyma [24]. Furthermore, its pathological mechanisms include multiple aging pathways, including telomere loss, epigenetic changes, altered nutrient sensing, mitochondrial dysfunction, cellular senescence, stem cell exhaustion, and chronic inflammation [25]. In nursing homes, residents with COPD are more likely to have comorbid heart failure than those without COPD [26].

It is necessary to address. Cancer is a clump of abnormal cells arising from normal cells, and cancer cells appear when two to ten genes of normal cells are damaged. Since this is induced gradually over a long period, aging plays a significant role in its development. The effects of aging have been noted in various cancers, including liver [27] and breast cancer [28]. The peak incidence of cancer is reported to be between 65 and 74 years of age, and the number of older adults diagnosed with cancer is expected to increase significantly with the aging of the population [29]. There are also reports that chronic psychological stress precipitates death from cancer [30]. On the other hand, there is concern about the increasing proportion of frail cancer survivors [31], and how to coexist with cancer is an important issue for an aging society.

In 2021, 537 million people worldwide were affected by diabetes. Furthermore, this number is expected to increase due to aging population [32]. Diabetes mellitus is a disease associated with end-organ damage, dysfunction, and failure of the retina, kidneys, nervous system, heart, and blood vessels. Furthermore, lifestyle influences the development of diabetes, especially type 2 diabetes. Aging has been pointed out as a factor that decreases the quality of life of diabetic patients [33]. Recently, research has also focused on the relationship between diabetes and age-related cognitive function. According to a large prospective study in the United States, women were more susceptible to diabetes-related cognitive decline with advancing age than men [34].

According to the National Institute on Aging-Alzheimer’s Association, Alzheimer’s disease assumes that β-amyloid deposition is an etiological factor. Furthermore, its neuropathological diagnosis is based on the coexistence of senile plaques (SPs) and neurofibrillary tangles (NFTs), which are designated as neuropathological changes of Alzheimer’s disease. NFTs are found in approximately 85% and 97% of individuals aged 65 and 80, respectively. Meanwhile, SPs occur in 30% and 50–60% at age 65 and 80, respectively. Alzheimer’s disease, a disease of the aging process [35], is the most common form of age-related dementia. It is characterized by progressive memory loss and cognitive impairment. Adult hippocampal neurogenesis (AHN) is known to decline rapidly in patients with Alzheimer’s disease [36]. Differences between urban and rural areas have been noted. Patients with dementia in rural areas have higher mortality rates, lower consultation rates, higher doses of antipsychotic medications, and lower use of home care services than those in urban areas [37].

Furthermore, a cohort study of an aging rural Japanese population found that the risk factors for decreased independence included hypertension, diabetes, being overweight, chronic kidney disease, current smoking, and history of stroke, heart disease, and cancer [38]. Stroke may be the greatest risk factor for decreased activities of daily living (ADL), represented by eating and toileting activities, and HRQOL, as it often results in hemiplegia. Chronic kidney and heart disease also reduce activity and inevitably worsen quality of life. Hypertension, diabetes, obesity, chronic kidney disease, smoking addiction, stroke, and heart disease are lifestyle-related diseases with causal mechanisms distinct to cellular senescence caused by aging, such as cellular and genetic changes described in the previous section. These diseases typically develop from high-calorie diets, lack of exercise, and excessive alcohol consumption; additionally, as life expectancy increases and such habits are prolonged, the risk of developing these diseases increases exponentially. In summary, the risk of developing lifestyle-related diseases increases with age unless lifestyle habits are changed [39,40,41].

The following section summarizes the impact of aging on HRQOL and reviews the relationship between these diseases.

## 4. Relationship between Aging, Disease, and HRQOL

Aging reduces mental and physical function and decreases the ability to perform activities of daily living and HRQOL. HRQOL is a useful outcome measure for treatment, especially in older patients whose physical and mental functions are difficult to improve once they have declined. In particular, the health state utility value is a centralized health-related quality of life score, with 0 indicating death and 1 indicating perfect health, so that scores can be compared between different diseases and across countries. Preference-based measures (PBMs) are typically used to measure the health state utility value. Of the many PBMs, the Euro QOL five-dimensional questionnaire (EQ-5D) is the most widely used PBM worldwide, because it has been translated into various languages and for which country-specific scoring algorithms are available. Several studies using the EQ-5D to study population norms in different countries have been published, as listed in Table 1. Although direct comparisons cannot be made because the country-specific algorithms used to calculate their respective EQ-5D scores are not identical, it is noticeable that the EQ-5D scores decline with age, regardless of country or race. Specifically, although the age ranges were not aligned because of the stated algorithmic differences between the studies, EQ-5D scores clearly declined from age 75 in Poland [42], 71 in Norway [43], 70 in Iran [44], 75 in Bulgaria [45], and over 75 in Japan [46]. This trend was consistent across regions and races.

Health data from Japan [46], which has the world’s oldest population, show a clear decline in HRQOL after the age of 80. Subjective symptoms, such as back pain, stiff shoulders, and arthritic pain, decreased their HRQOL. Interestingly, Yang et al. [48] found that in China, three aspects of the EQ-5D-5L were affected by aging: mobility, daily life, and anxiety/depression. In contrast, in a study of older adult subjects in European countries using the EQ-5D-3L [51], the scores decreased with age, but problems with pain and discomfort were most frequent (36–73%, any problems), and those with self-care practices were least frequent (3–31%, any problems). Next, we will review the association between typical age-related diseases and HRQOL.

Sensory organs play an important role in human life. This role does not change as we age, but unfortunately sensory function does decline.

The first of these is hearing loss. The impact of age-related hearing loss on HRQOL has been reported to cause decreased communication and social and emotional interactions [52]. Communication is an important function and means for connecting people. Even if the speech function is unaffected, smooth communication will be difficult if sound recognition is impaired. For hearing loss in older adults, hearing aids were reported to improve quality of life [53]. However, hearing aids are more expensive than eyeglasses; therefore, their cost should be considered.

Like hearing, vision is one of the most important senses in human life; it is used in communication, but more so for the enrichment and safety of one’s life. However, this function declines with age. In most cases, farsightedness can be compensated for by wearing graduated glasses. However, the impact of cataracts and glaucoma on HRQOL is more severe because these conditions cannot be improved by purchasing devices such as glasses. Tan et al. [54] reported that vision-related quality of life was significantly impaired in patients with cataract with high myopia. Furthermore, it was significantly improved after cataract surgery. Surgery for cataracts has also been reported to improve vision-related quality of life in both the first and second surgeries [55].

As people age, bone and joint deformities are more likely to occur owing to bone loss and wear-and-tear of cartilage and other tissues. Osteoarthritis of the knee joint is one such condition. A large Korean study reported higher odds ratios for depressed mood, psychological distress, and suicidal ideation, in addition to mobility impairment and pain in those with osteoarthritis [56]. The EQ-5D, a utility value scale, has also been shown to be effective in assessing the impact of changes in chronic pain related to osteoarthritis on HRQOL, even though it does not have excellent sensitivity or responsiveness for all diseases [57]. This study demonstrated that knee osteoarthritis sufficiently reduces HRQOL. In summary, osteoarthritis impairs mobility, increases pain, and causes psychological stress.

The relationship between COPD and quality of life was reported to be determined by specific factors, such as gender, severity index, pulmonary function parameters, body mass index, smoking, symptoms, complications, depression, anxiety, and exacerbations [58]. Lung function declines with age and this can facilitate the onset of COPD. Persistently poor lifestyle habits, such as obesity and smoking, can also contribute to the development of COPD. COPD decreases lung capacity, which consequently reduces the extent of activities in daily life, further diminishing HRQOL. A study that examined the effect of lung volume reduction surgery for COPD on HRQOL indices showed improvement in quality of life as measured by the 36-item Short Form Survey (SF-36) and EQ-5D [59].

Type 2 diabetes mellitus develops when a relative deficiency of insulin ensues from a genetic predisposition to insufficient insulin production, compounded by insulin resistance arising from an environmental predisposition to a poor lifestyle. In particular, an accumulation of fat in the liver and muscles due to lifestyle disorders, such as a high-fat diet, overeating, and a lack of exercise, reduces insulin function. A large European study demonstrated that frailty is more common in older adult diabetics, who tend to be depressed in addition to having low self-health ratings [60]. A study of a cohort of diabetic patients in China reported a correlation between cognitive decline and quality of life [61]. Thus, older adult patients with diabetes not only require insulin injections, but also experience a variety of adverse effects that persist throughout their lives and do not improve their HRQOL.

There have been numerous reports on the relationship between cancer treatment and HRQOL. This is because the side effects of chemotherapy have a significant impact on HRQOL. According to a study by Carelle et al. [62] the cancers most frequently affected by chemotherapy were breast, gastrointestinal, lung, and ovarian cancers, with the most severe side effects being “impact on family and partner”, the second most serious being “alopecia”, and the third most serious being “fatigue”. The impact of hormone therapy on HRQOL has also been reported [63]. Although targeted anti-human epidermal growth factor receptor 2 (HER2) agents have shown promising efficacy in older patients, it has been noted that the impact on HRQOL should be considered when extending treatment [64]. In any case, a HRQOL perspective is essential in evaluating cancer treatment in older adults.

One of the most worrisome health conditions associated with aging is dementia, typified by Alzheimer’s disease. Dementia is primarily characterized by negative symptoms such as memory and attention impairment. As the disease progresses, positive mental symptoms, such as delusions and personality disorders, gradually become dominant. A systematic review on the relationship between dementia and quality of life found that self-assessment by patients themselves was higher than that by caregivers or other proxies [65]. This is because as dementia progresses, the patient’s self-insight declines and they lack a sense of awareness of the disease. It is also important to assess the decline in caregivers’ quality of life. Furthermore, it has been found that caregivers’ quality of life declines as the patient’s severity of dementia worsens [66]. When discussing the decline in HRQOL due to dementia, it is as important to pay attention to and possibly measure the QOL of caregivers, such as spouses and children, because caring for dementia patients can be physically and emotionally demanding.

Heart disease and stroke are representative diseases caused by aging and lifestyle-related deteriorations. Both are caused by arteriosclerosis and hypertension, in which blood vessels narrow owing to deposits of cholesterol and other substances, resulting in poor blood flow. Cerebral infarction is caused by arteriosclerosis; however, in some cases, atrial fibrillation may result in a major cardiogenic cerebral embolism. When older people suffer from heart disease or have a stroke, their activity level decreases and their HRQOL declines. Golicki et al. [67] showed that the worst poststroke condition, as indicated by the Modified Rankin Scale, has a negative utility value. Although HRQOL does not decrease significantly in patients with cardiac disease, it is reported to be lower than that in healthy subjects [68]. Although some improvement can be expected with rehabilitation, these patients do not recover to a state of health as that before their illness. Therefore, a shift is being made in Japan from “medical treatment to cure”, which focuses on organ-specific treatment, to “medical treatment to cure and support”, which prioritizes treatment to maximize patients’ quality of life [69]. On the other hand, for these lifestyle-related diseases, not only have individual personality and temperament been noted as contributing influences, but also pollution of soil, water, air, and noise, as well as other environmental risks factors such as climate change, unhealthy urban design (e.g., lack of green space), unhealthy lifestyle habits, and psychosocial stress [70].

## 5. Discussion

Numerous studies have reported the relationship between aging and various diseases, and the reduced HRQOL they cause. Decline in HRQOL due to aging in various countries is particularly clear, as measured by the EQ-5D, a globally accepted utility value measure. Since humans are living organisms, aging occurs naturally through cell death and degeneration. These cellular changes lead to muscle weakness and joint damage that impede autonomy in daily life. Additionally, cellular degeneration occurs in nerve cells, leading to a decline in cognitive and mental functions that consequently causes a variety of problems in daily life.

Natural aging not only reduces HRQOL but also makes people more susceptible to various diseases. Although there are various causes for this, such as heredity, living environment, and lifestyle, this is a reality that occurs for most people.

Therefore, in an aging society, preventive measures are important to delay disease progression as much as possible. Here are some practical examples. There have been reports that long-term exercise programs for frail older adult residents of residential care homes prevented the increase in frailty [71] and that combined exercise and nutritional interventions were effective for older women with sarcopenia living in the community [72]. On the other hand, there are reports that vitamin D supplementation alone does not improve sarcopenia in community-dwelling older adults [73], so further results on nutritional supplementation are needed. Regarding lifestyle modification, there are reports that younger older adults are more motivated to make lifestyle changes to reduce their risk of dementia [74] and that healthy lifestyle modification programs, with particular emphasis on dietary changes, can help prevent CVD events, including myocardial infarction and stroke, in older adults living in the community [75].

In Japan, where the population is aging more than anywhere else in the world, there are measures to prevent long-term care [76]. They aim to maintain and improve physical, oral, and cognitive functions, many of which are implemented at the municipality level. These programs, held approximately once a week, are notable for their definitive effects on improving muscle strength in the upper and lower limbs and maintaining oral function [77]. Another notable topic is the introduction of robots that facilitate more efficient rehabilitation [78], communication with the older adults [79], and support for caregivers [80]. In Japan, efficiency is achieved through such technology in the face of a shrinking population.

Furthermore, since poor lifestyle habits such as unbalanced diets and lack of exercise are likely to cause diabetes, cardiovascular, and cerebrovascular diseases, efforts have been initiated to alter such habits. To encourage exercise, number of steps taken can be calculated and points can be awarded using digital devices, such as the Apple Watch. Virtual reality game applications also encourage individuals to improve their exercise habits; for example, Pokémon GO, a location-based augmented reality game for smartphones, has reportedly already improved exercise habits [81].

It is a positive sign that various municipalities and companies are providing services to mitigate the decline in HRQOL due to aging. The challenge to combat ageing has only just begun, and future developments are expected worldwide.

## 6. Conclusions

This article reviewed the effects of aging on human cells and the diseases that develop due to aging. In addition, the effects of these aging-related diseases on health are summarized from the perspective of HRQOL. Although human beings cannot resist aging, it is possible to improve and maintain HRQOL even after suffering from some diseases, and there will never be an end to calls for medical and healthcare programs that aim to achieve this goal. If this is the case, it is clear that assessment and outcome studies of HRQOL in the older adults will become increasingly important.

## Figures and Tables

**Table 1:** Comparison of the effects of aging in different countries using the EQ-5D.

First Author (Date)	Country	Study Participants	Mean EQ-5D Scores per Age Category	Measurement
Golicki (2015) [42]	Poland	3978	45–54: 0.898	EQ-5D-5L
			55–64: 0.856	
			65–74: 0.813	
			75+: 0.723	
McCaffrey (2016) [47]	Australia	2908	55–64: 0.89	EQ-5D-5L
			65–74: 0.87	
			75+: 0.83	
Stavem (2018) [43]	Norway	1131	51–60: 0.830	EQ-5D-3L
			61–70: 0.825	
			≥71: 0.785	
Yang (2018) [48]	China	650	50–59: 0.954	EQ-5D-5L
			60–69: 0.957	
			70+: 0.912	
Emrani (2020) [44]	Iran	3060	50–59: 0.75	EQ-5D-5L
			60–69: 0.74	
			70+: 0.67	
Encheva (2020) [45]	Bulgaria	1005	55–64: 0.914	EQ-5D-5L
			65–74: 0.876	
			75+: 0.789	
Shiroiwa (2021) [46]	Japan	10,185	50–59: 0.850	EQ-5D-5L
			60–69: 0.859	
			70–79: 0.826	
			80–89: 0.758	
Marten (2021) [49]	Germany	290	65–69: 0.92	EQ-5D-5L
			70–74: 0.85	
			75–79: 0.82	
			80+: 0.68	
Janssen (2021) [50]	European economies	21,425	45–54: 0.927	EQ-5D-3L
			55–64: 0.885	
			65–74: 0.865	
			75+: 0.785	

## Data Availability

The data presented in this study are available from the corresponding author upon reasonable request.
